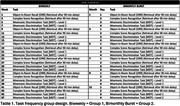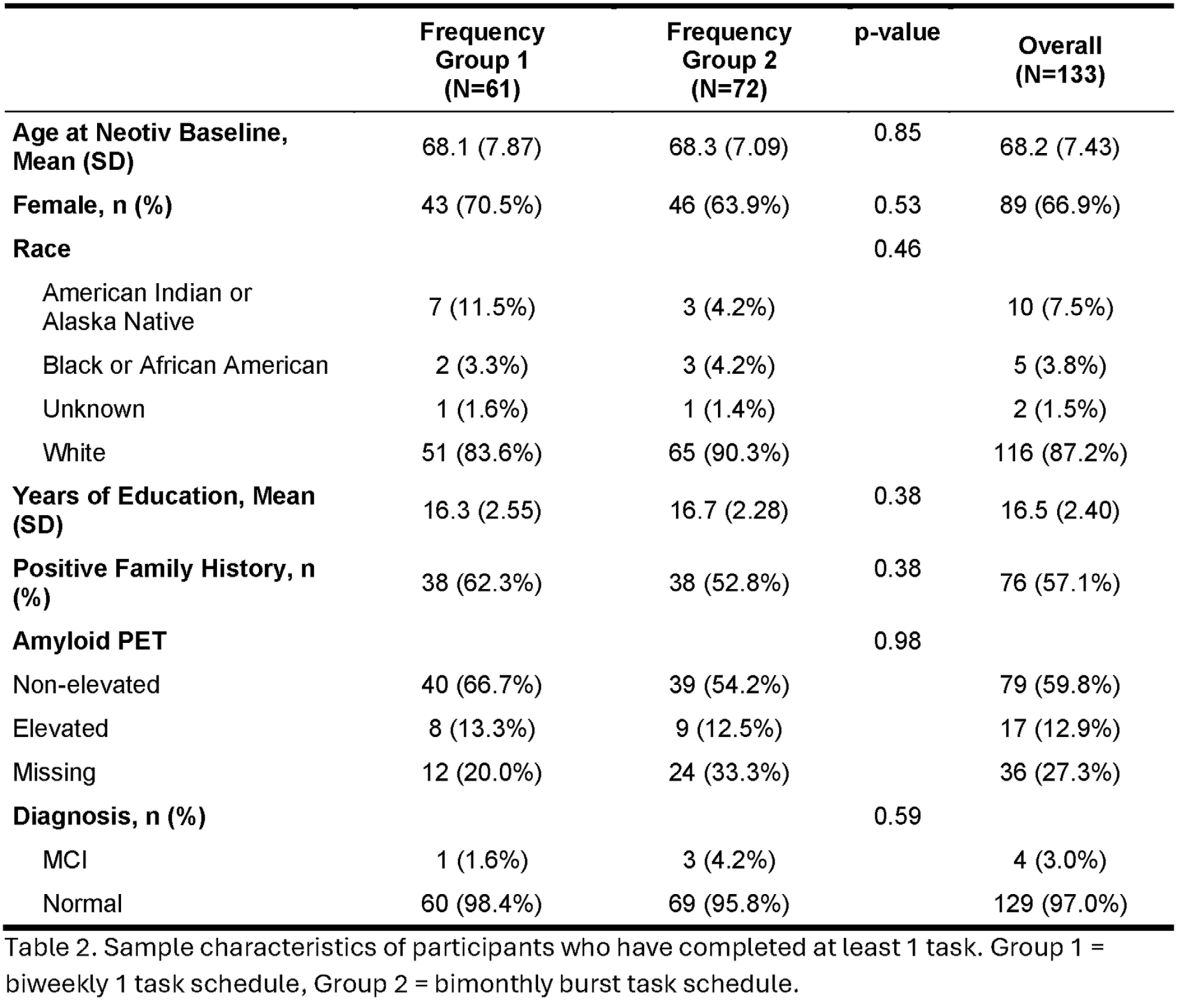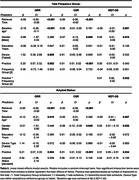# Procedural practice effects in remote digital memory assessment

**DOI:** 10.1002/alz.091794

**Published:** 2025-01-03

**Authors:** Kristin E Basche, David Berron, Amanda Peterson, Hannah Rosario, Sterling C. Johnson, Emrah Düzel, Lindsay R. Clark

**Affiliations:** ^1^ Department of Medicine, University of Wisconsin‐Madison School of Medicine and Public Health, Madison, WI USA; ^2^ Wisconsin Alzheimer’s Disease Research Center, University of Wisconsin‐Madison, School of Medicine and Public Health, Madison, WI USA; ^3^ Institute of Cognitive Neurology and Dementia Research (IKND), Otto‐von‐Guericke University, Magdeburg Germany; ^4^ Center for Behavioral Brain Sciences (CBBS), Magdeburg Germany; ^5^ German Center for Neurodegenerative Diseases (DZNE), Magdeburg Germany; ^6^ University of Wisconsin‐Madison School of Medicine and Public Health, Madison, WI USA; ^7^ Alzheimer’s Disease Research Center, University of Wisconsin‐Madison, Madison, WI USA; ^8^ Waisman Center, University of Wisconsin‐Madison, Madison, WI USA; ^9^ Geriatric Research Education and Clinical Center, William S. Middleton Memorial Veterans Hospital, Madison, WI USA; ^10^ Wisconsin Alzheimer’s Institute, University of Wisconsin School of Medicine and Public Health, Madison, WI USA; ^11^ Wisconsin Alzheimer’s Disease Research Center, Madison, WI USA; ^12^ University Hospital Magdeburg, Magdeburg Germany; ^13^ Wisconsin Alzheimer’s Disease Research Center, University of Wisconsin School of Medicine and Public Health, Madison, WI USA; ^14^ Wisconsin Alzheimer’s Institute, University of Wisconsin‐Madison School of Medicine and Public Health, Madison, WI USA

## Abstract

**Background:**

Frequent and remote cognitive assessment may improve sensitivity to subtle cognitive decline associated with preclinical Alzheimer’s disease (AD). However, repeated testing can result in unintended inflation of scores, due to practice effects. The objective of this study is to evaluate the extent of sessions with non‐identical stimuli on performance and determine if study design or amyloid PET status moderates the impact of practice.

**Method:**

Participants were recruited from longitudinal aging cohorts to complete medial temporal lobe‐based memory paradigms (Object‐In‐Room Recall [ORR], Mnemonic Discrimination for Objects and Scenes [MDT‐OS 1‐back and 2‐back], Complex Scene Recognition [CSR]) using the neotiv application at repeated intervals over one year. Participants were randomized to a task schedule (biweekly 1 task or bimonthly burst) for a total of 24 ten‐minute remote sessions. In both groups, participants completed each task 6 times; the interval between identical tasks was eight weeks (Table 1). Linear mixed effects models were used to assess the impact of practice on task performance and the interaction of practice with (1) task schedule group or (2) amyloid status. Models contained person‐level random intercepts and covariates of age, gender, education, and device type (tablet or smartphone).

**Results:**

Task schedule group did not differ by demographic characteristics (Table 2, n = 133, mean age = 68.2, 67% female, 87% white, 97% cognitively unimpaired). The interaction of practice x task schedule group was only significant for the MDT‐OS (Table 3, β = 0.01, p = 0.032). The main effect of practice was significant for the ORR (β = 0.20, p = 0.002) and CSR (β = 0.001, p<0.001) tasks. The interaction of practice x amyloid PET result was not significant in any models. The main effect of practice was significant in all models (Table 3, ORR: β = 0.21, p = 0.004; CSR: β = 0.01, p<0.001; MDT‐OS: β = 0.01, p = 0.034).

**Conclusion:**

Practice effects were present, indicating that procedural practice benefited task scores, despite non‐repeating stimuli. The MDT‐OS group x practice interaction indicated that participants in the burst design group had lower scores at their initial MDT‐OS visit, but increased performance with practice at a higher rate. No main effects or interaction of amyloid result was observed, possibly due to limited availability of data and few elevated participants.